# Water and Handwashing in a Drought-Prone Region of Southern Niger: How Environment, Household Infrastructure, and Exposure to Social and Behavior Change Messages Interact

**DOI:** 10.4269/ajtmh.22-0473

**Published:** 2023-02-06

**Authors:** Jessie Pinchoff, Leanne Dougherty, Chaibou Dadi

**Affiliations:** ^1^ Population Council, New York, New York;; ^2^ Population Council, Washington, D. C.;; ^3^ Conception Etudes Suivi Evaluation Appuis Formation, Niamey, Niger

## Abstract

This study aims to inform multisectoral development programs by exploring the extent to which social and behavior change (SBC) messages, environment, and household infrastructure are associated with knowledge and practice of handwashing behaviors. A cross-sectional survey of 2,708 households in the Maradi and Zinder districts of Niger was collected in April 2021. Household data were integrated with two local environmental measures: 1) water level at the nearest waterhole point, and 2) anomalous rainfall for the previous rainy season derived from climate hazards infrared precipitation with station rainfall (CHIRPS) data. Logistic regression models were constructed to explore how environment, household infrastructure, and exposure to SBC messages were associated with two hygiene-related outcomes: 1) observed water and soap available at household handwashing stations, a behavior, and 2) knowledge of critical moments for handwashing, a behavioral determinant. We find that in households near a water point with higher water depth, households were statistically significantly more likely (odds ratio [OR] = 1.25); (confidence interval [CI] = 1.12–1.49) to have water and soap observed at the handwashing station. Women in households near a water point with increased water depth (more water) were more likely to know three or more critical handwashing moments (OR = 1.07; CI = 1.03–1.11). Exposure to messages about the importance of handwashing was significantly associated with knowledge of critical handwashing moments and having water and soap observed at a handwashing station. Multisectoral programming should consider layering efforts so that development projects that increase access to water sources are complemented with SBC approaches focused on hygiene.

## INTRODUCTION

The Sahel region is marked by food insecurity, persistent poverty, and recurrent climate shocks that often drive vulnerable communities into crisis. Under-5 mortality rates are extremely high (84 per 1,000 in Niger),^1^ and Niger has the third-highest mortality rate for diarrheal disease in children under 5, linked with poor nutrition but also poor household water, sanitation, and hygiene (WASH) practices.^2^^,^^3^ Efforts to prevent diarrheal disease particularly in children include the promotion of handwashing with soap and water at key moments such as when preparing, serving, or eating meals, before feeding children, after using the toilet, or after disposal of feces, including changing children’s diapers.^4^ Despite these efforts, improvement has been slow, potentially due to droughts in the region and low access to water for many households.^5^

Social and behavior change (SBC) is a key strategy to promote and improve adoption of healthy behaviors, including proper hygiene. The goals are to increase knowledge, address attitudes and perceptions, and determine barriers to uptake of the behavior. Some SBC strategies may include promotion of health behaviors through mass media channels such as radio and community events, as well as through interpersonal communication with health providers and community volunteers. Although evaluations have shown that SBC approaches improve WASH outcomes,^6^^–^^8^ environmental conditions can lead to reduced effectiveness.^9^ For example, local drought reduces the availability of water to households, and households have to make tradeoffs regarding how water is used, for what purpose, and how frequently. In a region reliant on livestock and agriculture,^10^ water for handwashing and sanitation may not be prioritized when it is needed for drinking water, livestock, or supporting agriculture. Drought may result in migration for seasonal work and therefore influential audiences may not be present in the household or community to participate in SBC activities. And, finally, people may be selectively exposed or more likely to consider health promotion messages regarding handwashing when they have the environmental conditions and household infrastructure to support behavioral adoption.^11^

The United States Agency for International Development (USAID) Resilience in the Sahel Enhanced (RISE) initiative was developed in 2012 to strategically layer and sequence humanitarian assistance while reducing vulnerability to shocks, including drought, in regions of Burkina Faso and Niger over 5 years.^12^ In 2018, the USAID introduced RISE II with the aim of advancing the RISE initiative by incorporating best practices and lessons learned to address several key areas including elevating water security and focusing on targeted areas for joint action between health programs and infrastructure development aimed at increasing water availability. A critical component of RISE II was the introduction of an integrated SBC strategy that aimed to provide messaging across sectors to maximize contact with key audiences to promote a set of behaviors more efficiently. With the introduction of a multisectoral approach, the USAID aimed to leverage knowledge, expertise, reach, and resources from partners and sectors to benefit from their combined and varied strengths as they worked toward the shared goal of producing better health outcomes.

Previous research has found that behavioral determinants such as access to soap and perceived self-efficacy to practice handwashing are barriers to behavioral adoption.^13^ However, although there has been an increased focus on assessing how to best leverage multisectoral efforts, few studies have identified on how best to provide messaging relevant to several sectors.^14^ For example, peer group SBC activities link hygiene and nutrition messages to improve child health outcomes.^15^ However, these efforts could be amplified through collaboration with infrastructure programs by incorporating handwashing messages at new and established community water points. In addition, the effects of climate and drought on human health and behavior are not straightforward to measure, as drought is multidimensional and varies over time and space. Although the availability of improved measures of drought, including remotely sensed and satellite-derived indicators, has increased, many data sources on human health and behavior remain separate from climate, not allowing for an exploration of these linkages.^16^^,^^17^ Satellite imagery and remotely sensed data may provide opportunities to better understand how environmental conditions are associated with behavior and health outcomes, as they can be linked with survey data allowing for localized spatial and temporal patterns. Detailed information and data on the environment, including access to water, are critical to inform targeted hygiene activities, particularly ones that require water to carry out.^18^ Surveys with GPS coordinates can be integrated with environmental data using quantitative methods, thereby linking information that can be used to plan hygiene promotion activities. Localized rainfall measures and natural water source levels are two such measures available in the Sahel that are measured by satellites and can be linked with household surveys that assess household health behaviors. Direct questions in surveys regarding drought rely on perceptions of the respondent providing a subjective measure of water availability.^19^

This study aims to assess how three measures of drought relate to 1) household water availability at handwashing stations, a behavior addressed by SBC activities, and 2) knowledge of key handwashing moments, a behavioral determinant. It assesses factors that may be associated with this effect including household infrastructure, sociodemographic characteristics, and exposure to messages promoting handwashing to inform integrated SBC and multisectoral programming. It compares three measures of drought, one based on perception of drought, recent water levels nearby, and previous rainy season rainfall levels to understand how different aspects of drought may relate to handwashing outcomes.

## MATERIALS AND METHODS

### Study area and survey.

A quantitative survey was administered by trained staff in April 2021 with households in the Maradi and Zinder regions of southern Niger. Within each region, the USAID RISE II program implements multisectoral activities in 18 specific communes within five departments (administrative areas), led by three different RISE II resilience food security activities (RFSAs). For this study, 12 communes were sampled from comparison communes based on similar sociodemographic characteristics, population density, and healthcare accessibility. Maradi includes the city of Maradi, the second largest in Niger, but overall is quite rural. Zinder is very rural and extends north into the Sahel. Households in these districts rely on rainfed agriculture and pastoralism for their livelihoods.

We applied a three-stage stratified sampling procedure. In the first stage, we randomly selected six intervention communes from the 18 intervention communes and six comparison communes (four in Zinder and two in the Maradi region). In the second stage, we listed all enumeration areas (EAs) identified in the 2012 general census by commune in each of the randomly selected communes. We then used probability proportional to size to select EAs per commune starting at a random point and then systematically selecting areas using a fixed sampling interval. In total, we sampled 40 EAs for each group, stratified by commune. In the third stage, we enumerated all households in each of the randomly selected EAs with eligible women (married women between 15 and 49 years of age).

From households with eligible women, we randomly selected 34 households per EA to account for a 10% nonresponse rate and interviewed about 30 women between 15 and 49 years of age. A participant was randomly chosen (by flip of a coin) if the household had more than one eligible participant. Data collection was conducted by a Niger-based research partner, Conception Etudes Suivi Evaluation Appuis Formation, and administered in the local languages (e.g., Hausa). The Niger-based study team was composed of local interviewers fluent in the languages spoken in the regions. The study team informed participants that the survey included questions on personal and sensitive topics at the outset. They ensured participants they were free to terminate the interview at any point, and to skip any questions that they did not wish to respond to. They were also told that they may discontinue the interview at any time. The informed consent statement also included a statement requesting participant permission to share their deidentified data with other researchers and that their data would be made public in the aggregate.

We arrived at a final sample size of 2,708 women for analysis. This sample size is based on a minimum detectable difference for evaluation purposes of 6–11% points in the priority indicators (e.g., presence of a handwashing station at the household) between study groups, with 80% power to detect a difference and α = 0.05.

### Outcome measures.

Two outcome measures were used: water and soap or ash observed at the household handwashing station, and knowledge of the critical moments of handwashing as promoted by SBC programs. At each household, interviewers requested to observe the handwashing station used for an objective measure recorded at the time of the survey. Summary variables were created following the UNICEF Joint Partnership Monitoring guidelines.^20^ The presence of water and soap or ash was observed. Having both water and soap or ash observed at the handwashing station was the first key binary outcome, in reference to households who did not have a handwashing station or did not have a handwashing station with water and soap or ash. The second outcome was composed of an indicator regarding the five critical handwashing moments, and correctly identifying three or more of these moments. The five critical handwashing moments were before preparing a meal, before feeding others, before eating, after defecation, and after handling children’s feces.

### Measures of SBC exposure.

A binary measure for recent exposure to handwashing messaging was included in the survey (“In the last 3 months, have you heard or seen any messages about handwashing?”; yes or no) to capture exposure to handwashing promotion efforts through mass media channels such as radio and community events, as well as through interpersonal communication with health providers and community volunteers.^21^ Handwashing messages were primarily shared with women in local languages (Hausa and Kanuri).

### Measures of drought exposure.

We capture three distinct measures of water availability as an indicator for drought: perceived drought exposure (from the household survey), satellite-derived rainfall (using a gridded dataset from CHIRPS), and water levels at the nearest water point (remotely sensed water levels reported by GPS point). Each measure is analyzed separately because each assesses a different facet of drought, and each may relate to water availability at households at different time points. Logistic regression models were fit for each measure alone and then all three combined into a single final model.

First, survey participants were asked whether they had been exposed to drought in the last 12 months (“In the past 12 months has your village experienced a drought that is a lack of rainfall for a period of time that resulted in less available water than is required to meet the needs of your community?”). This variable characterizes perceived recent exposure to drought in the last 12 months as reported by each participant and is a subjective assessment dependent on the respondent’s experience and recall.

Separately, the GPS locations of households were overlaid with gridded, satellite-derived measures of rainfall as detected by climate hazards infrared precipitation with station data.^22^ Specifically, we included percent difference of rainfall from expected conditions for the rainy season just before the survey (the rainy season in Niger is June through August; the year prior to the survey is 2020). This is a gridded value of how much more or less rainfall was recorded compared with the median calculated since 1981. Climate hazards infrared precipitation with station data are available at a fine spatial resolution of 0.05° (∼5 km) for 1981 to the near present. Less rainfall than expected is used to characterize meteorological drought, important for various factors such as agriculture and household water access in a region such as Niger where people rely on rainfall. Throughout most of the year, there is no rain; over 95% of rainfall occurs during the rainy season.^23^ This period from June through August is therefore critical for agricultural productivity and local water resources that impact the full year. Rainfall for the week of the survey itself was 0 mm for all households; the previous rainy season is what is included in this analysis to assess recent rainfall and potential impacts on water availability.

Third, we used waterhole information from the Famine Early Warning Systems (FEWS) Water Point Monitor.^24^ The Water Point Monitor uses data from the Advanced Spaceborne Thermal Emission and Reflection Radiometer satellite to identify the location and surface areas of waterholes.^10^ Waterhole depth is generated daily, and we averaged this over the 1-week period prior to the survey date for each participant. The data are freely available through the FEWS Network (https://earlywarning.usgs.gov/fews/waterpoint/index.php) and are used to monitor food security and potential humanitarian aid needs.^10^ Using the GPS point of each of the households, we calculated the distance to the nearest waterhole point, assigning households the values for that location. Given there were only six waterhole points in the study area, multiple households could be assigned the same water point value. In models with a waterhole point, we include a fixed effect for the water point because many houses are clustered around each point.

### Sociodemographic variables.

We calculated a wealth tercile based on a series of survey questions regarding household asset ownership as well as the main drinking water source and type of toilet used by household members using Equity Tool methodology^25^ and also included women’s age. We assessed additional indicators, such as education level and marital status, but did not include them in the model due to collinearity.

Proximity to urban areas was also captured using distance to built-up areas (BUAs; an urban area is defined as a minimum building count of 13 for an area larger than 400,000 m^2^) and small settlement areas (any settlement extent that contains 50 or more buildings and is classified as a BUA) derived from GRID3 population and settlement estimates. The GPS for each surveyed household was used to quantify the distance in meters to the nearest BUA or residence within a BUA. Ultimately, a binary variable was created for urban or rural based on this variable. Lastly, we included a variable to denote which RFSA was working in the area where the household is located (Wadata, Hamzari, or Girma) because each conducted slightly different activities around handwashing and provision of water that may influence outcomes.

### Data analysis.

All variables of interest were tabulated to explore household demographic characteristics. Bivariate and multivariable logistic regression models were fit for each of the WASH outcome variables. Models were constructed for each WASH outcome variable to compare both household infrastructure and drought variables and assess exposure to SBC messages regarding handwashing adjusted for respondent age, wealth tercile, urban or rural location, and RFSA. In STATA, the standard errors were adjusted by strata and commune to account for the sampling approach. For the models that include water point data, we included a fixed effect for the water point they were closest to, because multiple households shared the same water point location. Separate models were constructed to highlight each of the three variables that assess water availability, to explore whether perception of drought, a measure of the recent rainy season, or current water availability in proximity to the household were associated with handwashing knowledge (a behavioral determinant) and with having water and soap at an observed handwashing station. A final iteration of the model includes all three because, although they are related, each captures a different dimension of drought.

### Ethics.

The Ministry of Public Health National Ethics Committee for Health Research in Niger provided approval for the study and consent forms (number 017/2020/CNERS). The study also received approval from the Population Council Institutional Review Board in the United States (protocol number 934). Study participants provided informed consent by marking the agreement (in the form of an X) without using their signature. This procedure is prevalent in similar study settings and allows for the inclusion of illiterate individuals or those without defined signatures.

## RESULTS

Table 1 presents descriptive statistics for the study sample. Among married women ages 15–49 in the study area, 50% were between the ages of 25 and 35 years old, and the majority (83%) lived in rural areas. A handwashing station with soap and water was observed in about 1 in 10 (11%) of all households sampled. Forty percent of women could identify three of five critical times for handwashing, whereas nearly two-thirds (64%) of women interviewed had heard or seen a message about handwashing in the 3 months preceding the survey. Almost half (49%) of women interviewed stated they had been exposed to drought in the past 12 months.

**Table 1:** Description of the RISE II study sample (*N* = 2,708 married women 15–49 years old)

Characteristics	Total	
	*N*	%
Women’s age		
15–24 years old	442	16.6
25–34 years old	1,334	49.9
35–49 years old	894	33.5
Household wealth terciles		
Poorest	913	33.7
Middle	893	33.0
Richest	902	33.3
Rural area (vs. urban)	2,259	83.4
Handwashing station with soap and water observed	221	8.2
Knowledge of three of five critical times for handwashing	1,083	40.0
In the last 3 months, have you heard or seen any messages about handwashing?	1,728	63.8
Resilience food security activities		
Wadata	804	29.7
Hamzari	953	35.2
Girma	951	35.1
Perceived drought in last 12 months	1,318	48.7
Average percent difference in rainfall for previous rainy season (June–September 2020) compared with median rainfall (mean, SD)	25.6	11.0
Average water point depth (mean, SD)	3.1	4.2

**Figure 1.**
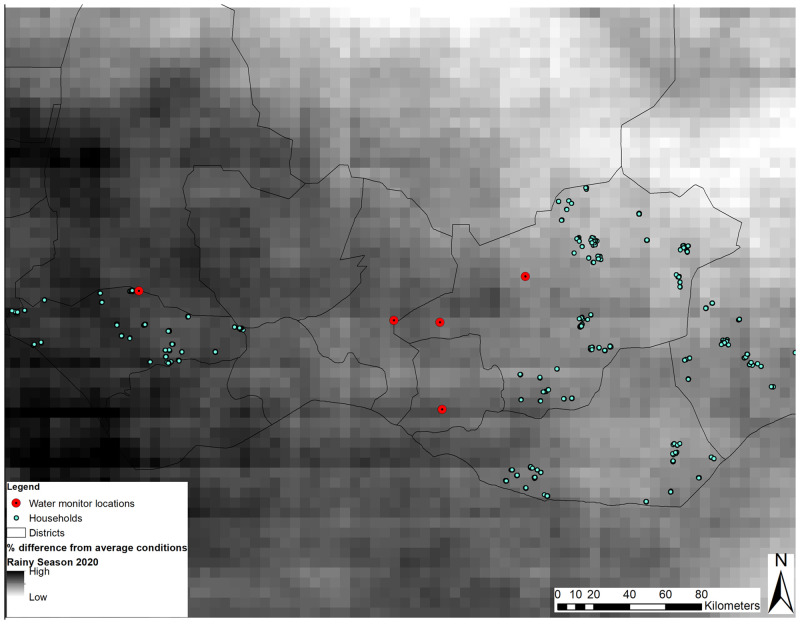
A map of the study area.

Figure 1 presents a map of the study area. GPS coordinates of households interviewed along with water point locations are indicated in the Niger study area. Household locations are jittered and thus not the exact location. Gridded percent difference in average rainfall is captured with lighter shading indicating less rainfall for the measurement period in reference to the previous year.

The multivariable logistic regression models for WASH outcomes are presented in Tables 2 and 3. In Table 2, factors associated with having water and soap or ash observed at the handwashing station are presented in four separate models that each test one of the three drought variables (perceived drought, rainfall anomaly, and waterhole depth) and one that includes all three drought variables. In the fully adjusted model, we find that women who reported perceived experience of a recent drought in the previous 12 months were statistically significantly less likely to have water and soap or ash at the handwashing station (odds ratio [OR] = 0.63; CI [0.45–0.88]) (model 4) compared with women who did not perceive a drought. As depth of water at the nearest water point increased (more groundwater available nearby), households were statistically significantly more likely to have water and soap or ash observed at their handwashing station (OR = 1.25; CI [1.12–1.49]) (model 4). For the previous rainy season, we see that households in areas that experienced more rainfall than expected during the 2020 rainy season were more likely to have water and soap or ash (OR = 1.04; CI [1.02–1.07]) (model 2) but this did not remain significant in the fully adjusted model. Households in the richest terciles were statistically significantly more likely to have water and soap or ash observed at the handwashing station in reference to households in the poorest tercile (OR = 2.59; CI [1.64–4.11]) in model 4. Finally, women who reported hearing a message on handwashing in the 3 months preceding the survey were statistically significantly more likely to have water and soap or ash observed at a handwashing station in all four models (OR = 2.78; CI [1.60–4.83]) (model 4).

**Table 2:** Factors associated with having water and soap or ash observed at the handwashing station (*N* = 2,670)

Variables	Model 1† OR (CI)	Model 2† OR (CI)	Model 3‡ OR (CI)	Model 4†‡ OR (CI)
Perceived drought	0.72* (0.50–1.01)	–	–	0.63*** (0.45–0.88)
Rainfall anomaly	–	1.04*** (1.02–1.07)	–	1.03* (1.00–1.07)
Water point depth	–	–	1.23*** (1.10–1.37)	1.25*** (1.12–1.39)
Wealth tercile (reference poorest tercile)	Reference	Reference	Reference	Reference
Middle tercile	0.89 (0.60–1.33)	0.96 (0.66–1.39)	0.89 (0.60–1.34)	0.86 (0.57–1.30)
Richest tercile	2.19*** (1.30–3.67)	2.31*** (1.36–3.94)	2.73*** (1.80–4.13)	2.59*** (1.64–4.11)
Rural (vs. urban)	1.19 (0.70–2.01)	1.04 (0.58–1.86)	1.76 (0.89–3.47)	1.87* (0.95–3.66)
Age category (reference 15–24 years old)	Reference	Reference	Reference	Reference
Age category (25–34 years old)	0.82 (0.54–1.25)	0.81 (0.54–1.22)	0.80 (0.50–1.27)	0.83 (0.53–1.29)
Age category (35–49 years old)	0.87 (0.49–1.53)	0.83 (0.46–1.47)	0.78 (0.45–1.35)	0.81 (0.48–1.40)
Heard message on handwashing	2.69*** (1.55–4.66)	2.67*** (1.57–4.54)	2.45*** (1.49–4.05)	2.78*** (1.60–4.83)

CI = confidence interval; OR = odds ratio. ***P < 0.01, **P < 0.05, *P < 0.1.

† Adjusted for resilience food security activities nearest the household.

**Table 3:** Factors associated with knowledge of three or more critical handwashing moments

Variables	Model 1† OR (CI)	Model 2† OR (CI)	Model 3‡ OR (CI)	Model 4†‡ OR (CI)
Perceived drought	0.51*** (0.42–0.63)	–	–	0.47*** (0.38–0.58)
Rainfall anomaly	–	1.01 (0.99–1.04)	–	0.99 (0.97–1.02)
Water point depth	–	–	1.06*** (1.02–1.10)	1.07*** (1.03–1.11)
Wealth tercile (reference poorest tercile)				
Middle tercile	1.56*** (1.20–2.02)	1.62*** (1.25–2.10)	1.55*** (1.20–2.00)	1.48*** (1.14–1.91)
Richest tercile	1.62*** (1.27–2.06)	1.68*** (1.25–2.10)	1.68*** (1.34–2.13)	1.62*** (1.27–2.07)
Rural (vs. urban)	0.92 (0.71–1.18)	0.83 (0.64–1.08)	0.84 (0.63–1.12)	0.93 (0.69–1.26)
Age category (reference 15–24 years old)				
Age category (25–34 years old)	0.75** (0.59–0.96)	0.74** (0.58–0.94)	0.71** (0.56–0.90)	0.71*** (0.56–0.91)
Age category (35–49 years old)	0.83 (0.65–1.06)	0.78* (0.61–1.00)	0.72** (0.56–0.92)	0.76*** (0.59–0.97)
Heard message on handwashing	1.39*** (1.16–1.68)	1.34*** (1.13–1.60)	1.32*** (1.11–1.58)	1.38*** (1.14–1.68)

CI = confidence interval; OR = odds ratio. ***P < 0.01, **P < 0.05, *P < 0.1.

† Fixed effect for commune.

‡ Adjusted for nearest water point.

In Table 3, factors associated with knowledge of three or more critical handwashing moments are presented for each of the three drought measures (perceived drought, rainfall anomaly, and waterhole depth) in models 1, 2, and 3 and a combined final model 4. We find that women who reported perceived experience of drought in the previous 12 months were statistically significantly less likely to know three or more critical handwashing moments (OR = 0.47; CI [0.38–0.58]) (model 4). As water depth increased at the nearest water point (more groundwater available), women were more likely to know three or more critical handwashing moments (OR = 1.07; CI [1.03–1.11]) (model 4). Households with higher rainfall in the previous rainy season compared with average conditions were not statistically significantly more likely to know three or more critical handwashing moments. Households in the middle and richest terciles in reference to households in the poorest tercile were statistically significantly more likely to know at least three of the five critical moments for critical handwashing, in all iterations of the model. No difference was observed for rural versus urban households. Older women had lower odds of knowing at least three critical handwashing moments compared with women 15–24 years old (OR = 0.76; CI [0.59–0.97]) (model 4). Finally, women who reported hearing a message on handwashing in the 3 months preceding the survey were statistically significantly more likely to know at least three critical handwashing moments in all models (OR = 1.38; CI [1.14–1.68]) (model 4).

## DISCUSSION

Multisectoral coordination provides an opportunity to leverage the strengths of various stakeholders and sectors to jointly achieve a development outcome. Multisectoral programs are increasingly becoming of interest as global health programs address complex challenges such as climate change. In this study, we assessed the association of three measures of drought with household water and soap availability at handwashing stations and knowledge of key handwashing moments to inform an integrated SBC and multisectoral program. We also assessed factors that may mitigate this effect including household infrastructure, sociodemographic characteristics, and exposure to messages promoting handwashing. We found that exposure to messages about the importance of handwashing was significantly associated with knowledge of critical handwashing moments and household water and soap availability at handwashing stations, highlighting the critical importance of multisectoral coordination. SBC campaigns are an important and effective tool in increasing knowledge of healthy behaviors but, in a climate-stressed setting like Niger, the ability to act on this knowledge may be limited by water availability. In this study, environmental characteristics, including exposure to drought, were significantly associated with a household’s ability to adopt hygiene-related behaviors. Consistent with previous studies, household infrastructure and wealth measured through a wealth index was an important factor in having water and soap at the handwashing station.^26^^,^^27^ Taken together, our findings suggest that household infrastructure and water access challenges in this drought-prone setting should be considered when addressing hygiene-related behavior.^28^ In particular, there should be an increased emphasis on addressing the needs of economically vulnerable groups through the development of, for example, pro-poor sanitation business models.^29^

Findings from this study help to further build the evidence base on the importance of aligning SBC programs with multisectoral activities and emphasize the need to work collaboratively on structural determinants (e.g., expanding access to climate-sensitive WASH infrastructure). Previous studies have found that SBC approaches are effective but disruptions to water supply reinforce the need to implement multisectoral approaches that can address infrastructure and behavioral outcomes at the same time.^30^^,^^31^ Public health programs should also consider alternative handwashing methods. Several studies have highlighted methods used by local communities for decontamination purposes such as alkaline (ash), salt, and sand as “dry tap” options.^32^ One study suggests alcohol-based hand sanitizer can improve hand hygiene in water-constrained environments and can be promoted to complement existing soap and water-based handwashing messages.^33^ SBC approaches can also further multisectoral efforts by building awareness and capacity of government stakeholders to mainstream climate change adaptation into multisectoral planning processes.^34^

The Sahel region has long experienced drought, but this will worsen in the future.^35^ Climate projections indicate that rainfall will become more intense, unpredictable, and less frequent, with impacts on the frequency and intensity of major droughts.^12^ Meanwhile, average temperatures in the region will continue to increase. Although the Sahel region contributes little to greenhouse gas emissions, its temperatures are rising 150% faster than the global average.^36^ Given the increasing role that climate change is having in health outcomes, there is a need to further develop and incorporate environmental measures into ongoing health-related studies to better understand how climate change is affecting health outcomes as well as to provide monitoring tools to respond to health emergencies. For example, monitoring rainfall patterns can serve as an early warning system for diarrheal prevalence and the need to reinforce hygiene programs.^37^ Early warning systems can be used to advocate for increased action planning related to maintaining a designated place for handwashing with sufficient soap and water, which has previously been shown as an effective handwashing behavioral determinant.^38^

### Limitations.

Several important limitations should be acknowledged with respect to this study. Measurement of handwashing behaviors is challenging because without direct observation during critical moments, study teams are faced with self-reported measures where study participants may be more likely to report practicing the behavior when they regularly do not due to social desirability bias.^39^ To address these challenges, the study team relied on both observation and self-reported measures related to knowledge of handwashing practices to capture handwashing behavior.^40^ Research has shown that increasing knowledge related to handwashing behaviors is associated with increased handwashing behavior.^27^^,^^41^ A second limitation from this study is that there were a limited number of water points in the study area to draw from and so the water point data were considered a proxy for the nearest source. Previous studies have found that preferred water sources may vary depending on seasonal availability. Our study is unable to confirm that the satellite-derived water point reflects the levels of water at the preferred source at the time of the study.^42^^,^^43^ Finally, although we have assessed exposure to handwashing messages, the study did not explicitly capture exposure to other activities such as distribution of handwashing stations. However, we have included a variable to adjust for implementation partners, which may account for variations in implementation approaches.

## CONCLUSION

Public health problems are complex and, in many cases, a single health issue may be influenced by interrelated social, environmental, and economic factors that can best be addressed with a holistic, multisectoral approach. Program activities that focus on improving hygiene-related behaviors through SBC approaches should continue to use these approaches while also recognizing the importance of integrating infrastructure improvements that address water shortages that may be limiting people’s abilities to act upon knowledge gained through these SBC approaches. Similarly, multisectoral programming should consider layering efforts so that development projects that increase access to water sources are complemented with SBC approaches focused on hygiene and sanitation. Future health-related studies in drought-prone settings should also consider incorporating environmental measures to strengthen our understanding of how climate change influences health outcomes and behaviors.
